# Enhanced purification coupled with biophysical analyses shows cross-β structure as a core building block for *Streptococcus mutans* functional amyloids

**DOI:** 10.1038/s41598-020-62115-7

**Published:** 2020-03-20

**Authors:** Ana L. Barran-Berdon, Sebastian Ocampo, Momin Haider, Joyce Morales-Aparicio, Gregory Ottenberg, Amy Kendall, Elena Yarmola, Surabhi Mishra, Joanna R. Long, Stephen J. Hagen, Gerald Stubbs, L. Jeannine Brady

**Affiliations:** 1 Department of Oral Biology, University of Florida, Gainesville, Florida USA; 2 Department of Biological Sciences and Center for Structural Biology, Vanderbilt University, Nashville, Tennessee USA; 3 Department of Physics, University of Florida, Gainesville, Florida USA; 4 Department of Biochemistry, University of Florida, Gainesville, Florida USA

**Keywords:** Biophysics, Chemical biology, Microbiology, Structural biology

## Abstract

*Streptococcus mutans* is an etiologic agent of human dental caries that forms dental plaque biofilms containing functional amyloids. Three amyloidogenic proteins, P1, WapA, and Smu_63c were previously identified. C123 and AgA are naturally occurring amyloid-forming fragments of P1 and WapA, respectively. We determined that four amyloidophilic dyes, ThT, CDy11, BD-oligo, and MK-H4, differentiate C123, AgA, and Smu_63c amyloid from monomers, but non-specific binding to bacterial cells in the absence of amyloid precludes their utility for identifying amyloid in biofilms. Congo red-induced birefringence is a more specific indicator of amyloid formation and differentiates biofilms formed by wild-type *S. mutans* from a triple ΔP1/WapA/Smu_63c mutant with reduced biofilm forming capabilities. Amyloid accumulation is a late event, appearing in older *S. mutans* biofilms after 60 hours of growth. Amyloid derived from pure preparations of all three proteins is visualized by electron microscopy as mat-like structures. Typical amyloid fibers become evident following protease digestion to eliminate non-specific aggregates and monomers. Amyloid mats, similar in appearance to those reported in *S. mutans* biofilm extracellular matrices, are reconstituted by co-incubation of monomers and amyloid fibers. X-ray fiber diffraction of amyloid mats and fibers from all three proteins demonstrate patterns reflective of a cross-β amyloid structure.

## Introduction

Amyloids are ordered protein aggregates with similar quaternary structures and biophysical characteristics. They are formed when soluble proteins change conformation and self-assemble into insoluble fibrillar aggregates^1^. Amyloids were initially identified in the context of pathology but functional amyloids are now recognized in all kingdoms of life, mediating desirable activities for the organisms that produce them^2,3^. Microbes use amyloids for their mechanical and biological properties^4^. Amyloid fibers have a tensile strength comparable to steel^5^, and are protease and detergent resistant^6–8^. Multiple bacterial functional amyloids have been identified that can contribute to adhesion, biofilm development, genetic competence, cell density regulation, host interactions, and/or aerial hyphae formation^9–12^. Microbial amyloid formation is generally recognized as a biofilm-associated process^13–15^. *S. mutans* is a major causative agent of human dental caries, a classic biofilm related disease^16^. Proteins that assemble into amyloid can serve as scaffolds to increase stiffness and unify the extracellular matrix (ECM) and cells within biofilms^11,17,18^. Bacterial amyloids also participate in other biological processes. For example, Fap amyloid fibrils of *Pseudomonas aeruginosa* can serve as a functional sink for quorum-sensing molecules^19^. While bacterial amyloids share many common features, unique species-specific aspects are also being identified.

Amyloids display distinct tinctorial properties and spectral shift upon uptake of dyes such as Thioflavin T (ThT) that increase in fluorescence intensity upon binding. When Congo-red (CR) binds to amyloid it becomes birefringent due to its orientation and optical properties, visualized as anomalous yellow-green or orange colors under crossed-polarizers^20,21^. Amyloids display a stable assembly of β-sheets packed perpendicular to the fiber axis when examined by X-ray diffraction, Fourier-transform infrared spectroscopy and solid-state nuclear magnetic resonance^22,23^. Amyloid fibers vary in length but display typical diameters of 4–11 nm6. X-ray fiber diffraction has identified a stacked cross β-sheet quaternary structure of amyloid fibers with a common diffraction pattern of ~4.7–4.8 Å meridional signals, corresponding to the distance between hydrogen-bonded β-strands, and more diffuse ~10–12 Å equatorial signals arising from association of the sheets^24^. Additionally, the fiber structure of the phenol-soluble modulin α3 peptide of *Staphylococcus aureus* is reported as an atypical “cross-α“ amyloid-like architecture with amphipathic α-helices stacked perpendicular to the fiber axis in self-associating sheets^25^. Thus, it is important to characterize individual bacterial amyloids to identify both shared and novel features.

A paradigm is emerging for Gram-positive organisms whereby surface-localized proteins serve dual functions as adhesins, and are also processed into amyloidogenic derivatives that can polymerize depending on prevailing environmental conditions^13,17^. *Streptococcus mutans* produces a typical biofilm ECM composed of polysaccharides, proteins, and eDNA. Amyloid formation is a biofilm-related event in *S. mutans* with three β sheet-rich amyloid-forming proteins identified to date^26^. P1 (aka AgI/II) and wall-associated protein A (WapA) proteins are surface-localized sortase substrates whose truncation derivatives, C123 (aka, AgII) and AgA, are amyloidogenic. Transpeptidase sortase enzymes covalently link their substrates to the cell wall peptidoglycan^27^. *S. mutans* lacking sortase is defective in amyloid production suggesting a potential amyloid nucleation mechanism at the cell surface^28^, analogous to the process described for *E. coli* curli amyloid formation^15,29,30^. Smu_63c, a third amyloidogenic protein identified in *S. mutans*, is a secreted protein that appears to serve as a negative regulator of genetic competence and biofilm cell density^26,31^. All three of these *S. mutans* proteins influence biofilm development, which is inhibited by known inhibitors of amyloid fibrillization such as tannic acid and epigallocatechin gallate^26^.

The purpose of this study was to evaluate methods to study *S. mutans* amyloid formation *in vitro*, to further characterize *S. mutans* amyloids, and to track the dynamics of amyloid formation *in situ* within growing biofilms. The new information described herein will facilitate the design of future experiments to evaluate environmental conditions that influence amyloid formation of *S. mutans* proteins *in vitro* and within biofilms.

## Results

### Purification and characterization of amyloid fibers

SDS-Page and Western blot confirmed elimination of protein monomers from each induced amyloid sample by treatment with proteinase K (PK) or PK/Triton. (Fig. S1). All samples showed a different solubility before and after treatment. Prior to amyloid induction, proteins remained in solution. After induction but before protease digestion, amyloid material remained in a homogenous suspension, but once monomers were eliminated the material fell to the bottom of the tubes (Fig. S2). Each protease treated sample was lyophilized and weighed to quantify recovered protease-resistant amyloid material, ~10–20% of the total starting amount of each protein. Insoluble protease-resistant amyloid was suspended in ddH_2_O by vortexing and small cycles of sonication for further physicochemical characterization.

### Transmission electron microscopy

**Figure 1:**
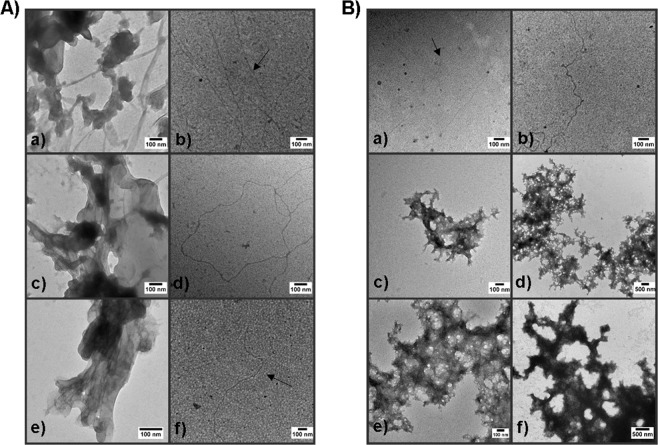
Transmission electron microscopy (TEM) of amyloid mats and purified fibers (**A**) TEM images of *S. mutans* amyloid before and after removal of residual monomer. a, c and e) Induced amyloid produced from purified C123, AgA, and Smu_63c, respectively. b, d and f). Amyloid material produced from C123, AgA, and Smu_63c following proteinase K digestion. (**B**) TEM of purified C123 amyloid fibers incubated without stirring with and without added monomers. a, c, and e) Purified C123 fibers incubated for 2 weeks alone (a) or with added C123 monomer at final concentrations of 1% (c) or 10% (e). e, d, and f) Purified C123 fibers incubated for 4 weeks alone (b) or with added C123 monomer at final concentrations of 1% (d) or 10% (f).

Crude induced amyloid and residual insoluble material harvested after protease digestion were visualized by TEM. Induced amyloid of each protein appeared as mat-like aggregates (Fig. 1A panels a, c, e), consistent with previously published images of purified bacterial amyloid proteins produced by *S. mutans* as well as by *Bacillus subtilis*^8,26,32^, and similar in appearance to nanocomposite structures comprised of amyloid fibers and graphene^33^. Sample morphologies were notably different following protease digestion in which typical amyloid fibers with diameters of ~4 nm remained (Fig. 1A panels b,d,f). We speculated that the mat-like structures may represent a composite of amyloid fibers and randomly aggregated monomers. Purified fibers of C123 were therefore incubated at 37 °C for 2 or 4 weeks without stirring in the presence of 1 or 10% (w/w) added monomer (Fig. 1B). Fibers without monomer were included as a negative control. The C123 fiber only controls showed no change in appearance over time, but the samples with added monomer reassembled into mat-like structures.

### Fluorescent dye assays

**Figure 2:**
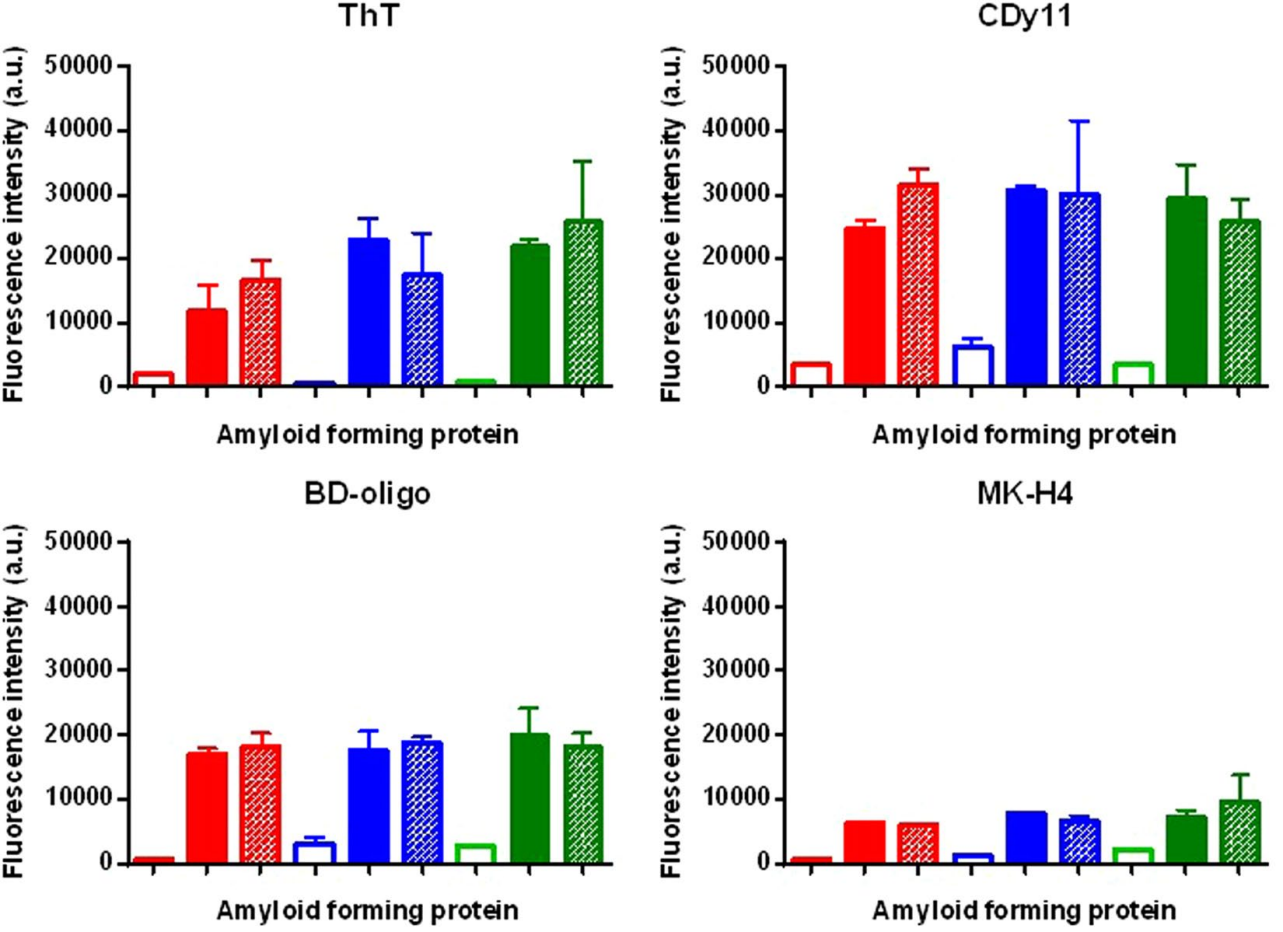
Amyloidophilic dye uptake assays. Ten micrograms of purified recombinant C123 (red bars), AgA (blue bars), or Smu_63c (green bars) were reacted with ThT, CDy11, BD-oligo, and MK-H4. Empty bars correspond to the purified monomeric proteins, solid bars to the samples after amyloid induction, and diagonal patterned bars to the purified amyloid fibers after treatment of the amyloid material to remove residual monomers. C123 and AgA polypeptides correspond to naturally occurring amyloidogenic derivatives of P1 and WapA, respectively. Arbitrary units (a.u.) ThT (Excitation = 440, Emission = 485), CDy11 and BD-Oligo (Ex = 530, Em = 580), and MK-H4 (Ex = 475, Em = 567). Protein concentration 0.1 mg/mL. Error bars represent standard deviations.

In addition to ThT^34^, newer amyloidophilic dyes have also been reported. We performed dye uptake assays with ThT, CDy11^35^, BD-oligo^36^ and MK-H4 (Young-Tae Chang, personal communication), comparing protein monomers to induced amyloid material and purified protease resistant amyloid fibers (Fig. 2). All dyes readily distinguished between monomeric and both amyloid forms of each protein. Fluorescence values were highest for CDy11, followed by ThT and BD-oligo. MK-H4 was the least sensitive dye tested.

### Congo red birefringence

**Figure 3:**
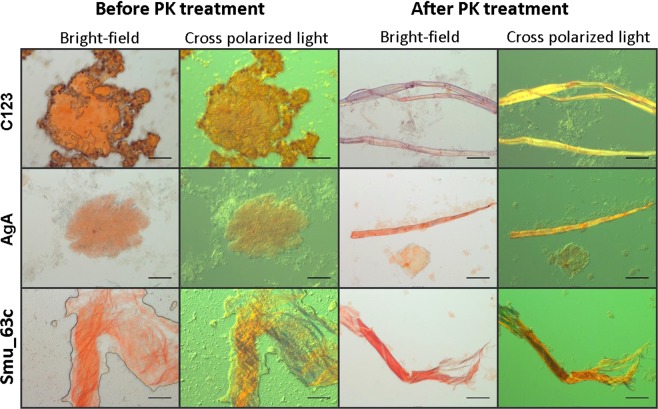
Congo red-induced birefringence of amyloid material before and after treatment with proteinase K (PK). Purified recombinant Smu_63c, and recombinant C123 and AgA polypeptides, which correspond to naturally occurring amyloidogenic derivatives of P1 and WapA respectively, were evaluated. Samples were visualized by bright-field microscopy and under crossed polarizing light filters. Scale Bar 50 µm.

When induced amyloid and purified fibers of each protein were stained with CR, all samples demonstrated typical birefringent properties (Fig. 3). Prior to protease treatment samples looked mat-like and birefringence was more diffuse. After protease treatment birefringence was more pronounced and rope-like structures were visualized. Protein monomers did not stain with CR and were not visible by light microscopy (not shown).

### Protein tertiary structure and X-ray fiber diffraction

**Figure 4:**
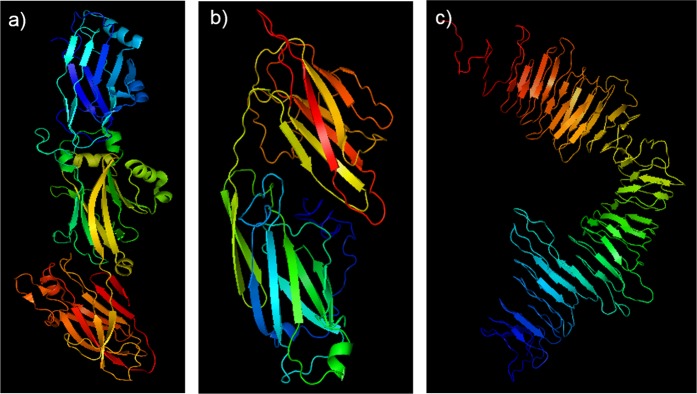
Schematic representation of the solved or predicted tertiary structures of P1-C123 (**a**), WapA-AgA (**b**), and Smu_63c (**c**). The illustration of C123 is based on the solved crystal structure^37^ (PDB id:3QE5). The predicted structures of AgA and Smu_63C were determined using I-TASSER^38^. Blue to red rainbow coloring indicates progression from N- to C-termini.

**Figure 5:**
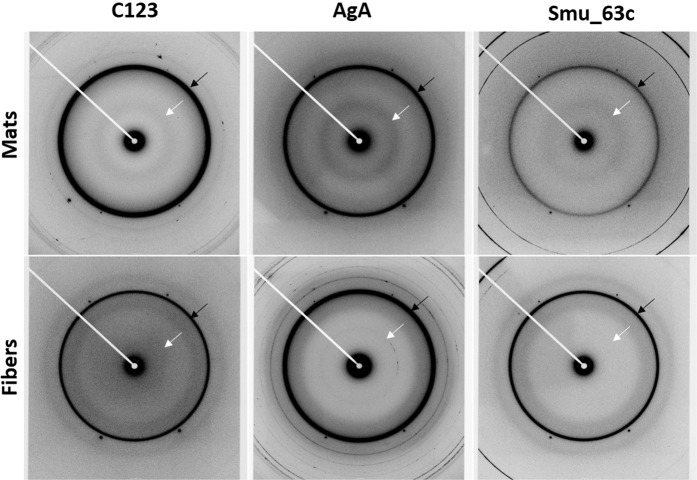
X-ray fiber diffraction patterns of amyloid mats and purified amyloid fibers derived from P1-C123, WapA-AgA and, Smu_63c. Black arrows indicate meridonal diffraction at ~4.8 Å. White arrows indicate equatorial diffraction at ~10.5 Å.

The crystal structure of C123 has been solved and is comprised of three domains (C1–3), each adopting a DE-variant IgG fold with two beta sheets linked through isopeptide bonds^37^ (Fig. 4, panel a). Crystal structures of AgA and Smu_63c are not known. We utilized I-TASSER^38^ to predict structures of AgA and Smu_63c (Fig. 4, panels b and c). AgA appears as two beta sheet-rich domains connected by disordered loops. Smu_63c also appears rich in beta sheet structure. While all three proteins exhibit predominantly beta sheet structure, the domain organization of each is distinct and no primary sequence homology is apparent (Fig. S3). We evaluated X-ray diffraction patterns of amyloid mats and purified amyloid fibers of C123, AgA and Smu_63c (Fig. 5). Common features included diffraction rings detected at ~4.8 Å as well as at ~10.5 Å. These signals are in good agreement with reported meridional reflections at ~4.7–4.8 Å that stem from β-strands stacked perpendicular to the fiber axis, and equatorial reflections at ~10–12 Å on the equator that stem from β-sheets running parallel to the fiber axis^39^. Observed rings, rather than separated reflections, are consistent with diffraction of non-aligned fibers in the samples.

### Detection of *S. mutans* amyloid within biofilms in a microfluidics flow cell

**Figure 6:**
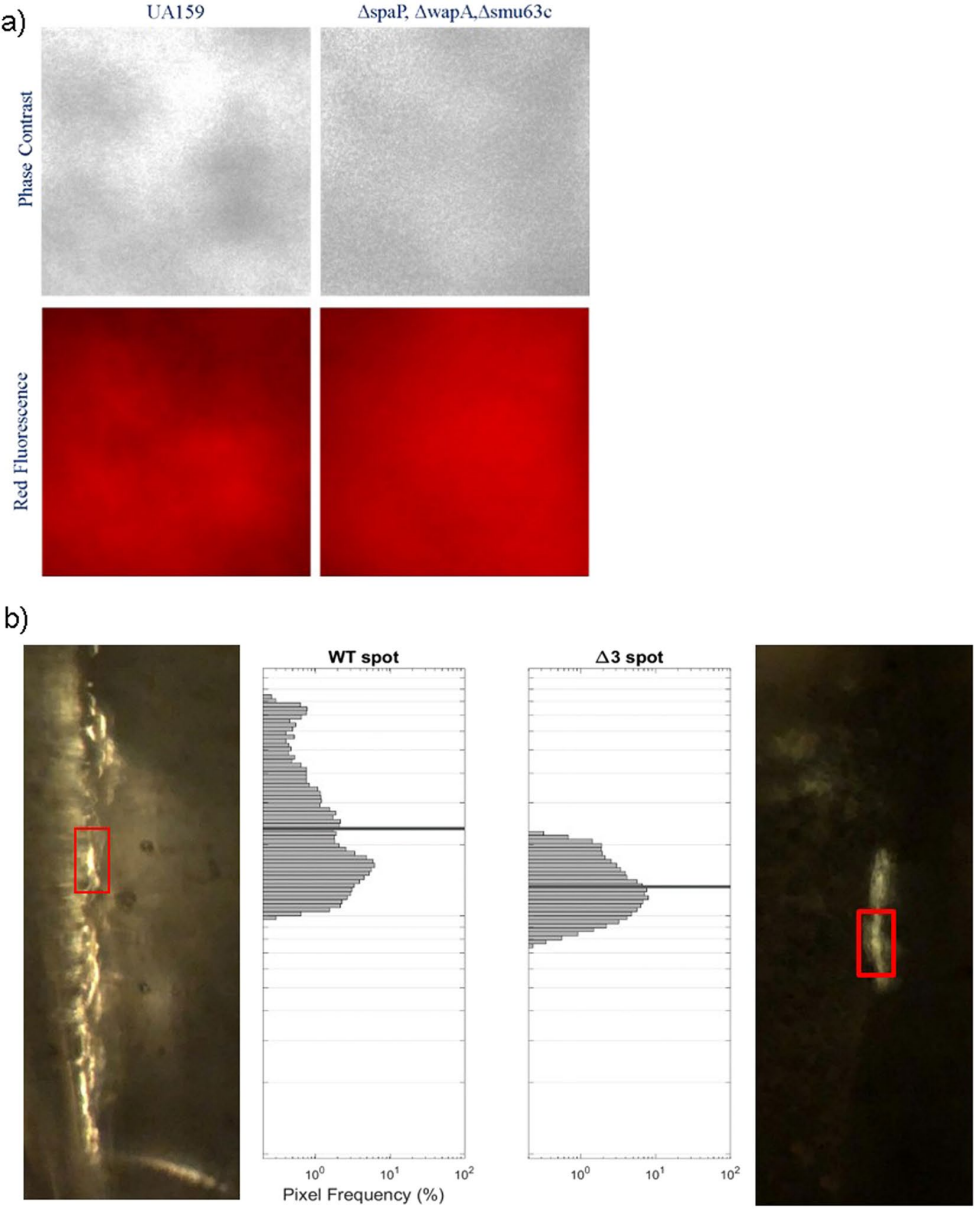
Assessment of amyloid formation during *S. mutans* biofilm growth in a microfluidics system. (**a**) Phase contrast and red fluorescence images following staining of 72 h biofilms of *S. mutans* wild-type and a triple mutant (Δ3) lacking P1 (encoded by *spaP*), WapA, and Smu_63c with CDy11. Similar patterns of dye binding to both strains suggests CDy11 reactivity with non-amyloid material. (**b**) CR-induced birefringence of 72 h biofilms of *S. mutans* wild-type (left) and the Δ3 triple mutant (right). Insets indicate pixel frequency of the brightest visual locations (red boxes) in each image. In contrast to staining with CDy11, biofilms of the WT and mutant strains could be differentiated on the basis of CR-induced birefringence.

To evaluate amyloid formation *in situ* within growing biofilms we compared the *S. mutans* wildtype and a triple deletion (Δ3) mutant^26^ growing in microfluidic channels under steady flow of medium. We utilized CDy11 since it was the most sensitive dye for detecting amyloids in the *in vitro* assay (Fig. 2). First, we confirmed that CDy11 could discriminate monomeric from amyloid protein forms by fluorescent microscopy (Fig. S4). Then *S. mutans* cells were grown in glass capillaries under steady flow for 72 h, exposed to CDy11 and imaged in phase contrast and fluorescence microscopy (Fig. 6, panel a). Bacterial biofilms were evident on the capillary walls of both strains, and CDy11 staining paralleled bacterial accumulation in the chambers over time (not shown), but the strains did not differ in red fluorescence following CDy11 staining. The absence of differential staining between wild-type and mutant strains suggested non-specific background binding of the dye to non-amyloid material in the biofilms. Thus, a potentially more discriminating method for tracking amyloid formation *in situ* was investigated. *S. mutans* biofilms were grown in capillaries as described above, but exposed to CR and visualized by bright-field microscopy and under crossed polarizers. CR-induced birefringence was more prevalent and of higher measurable intensity in the wildtype compared to the Δ3 mutant strain (Fig. 6, panel b). Residual CR-induced birefringence in the Δ3 mutant strain biofilm suggests that additional unidentified amyloidogenic proteins may also be present. Further, stronger birefringence was observed with the polarizing axis at 45° with respect to the direction of media flow, than with parallel or perpendicular alignment. The Congo red molecule is believed to bind to amyloid with its electric dipole perpendicular to the long axis of the fibril^40^. Therefore the maximum Congo red birefringence is expected when the polarizer is aligned at 45° degrees relative to the fibril axis^41^. Our data therefore suggest that the amyloid fibers are at least partially aligned along the flow direction. We also investigated whether non-specific binding to *S. mutans* was problematic for ThT. Bacteria were cultured under planktonic conditions in which amyloid production would be minimal^8,9^. In addition, reactivity of ThT with cells from the wildtype strain was compared to that of the Δ3 and Δ*srtA* mutants in which biofilm-associated amyloid formation is impaired^26,28^. Similar ThT associated fluorescence was observed for all three strains and was directly proportional to cell number (Fig. S5).

### Time course of amyloid formation in static biofilms

**Figure 7:**
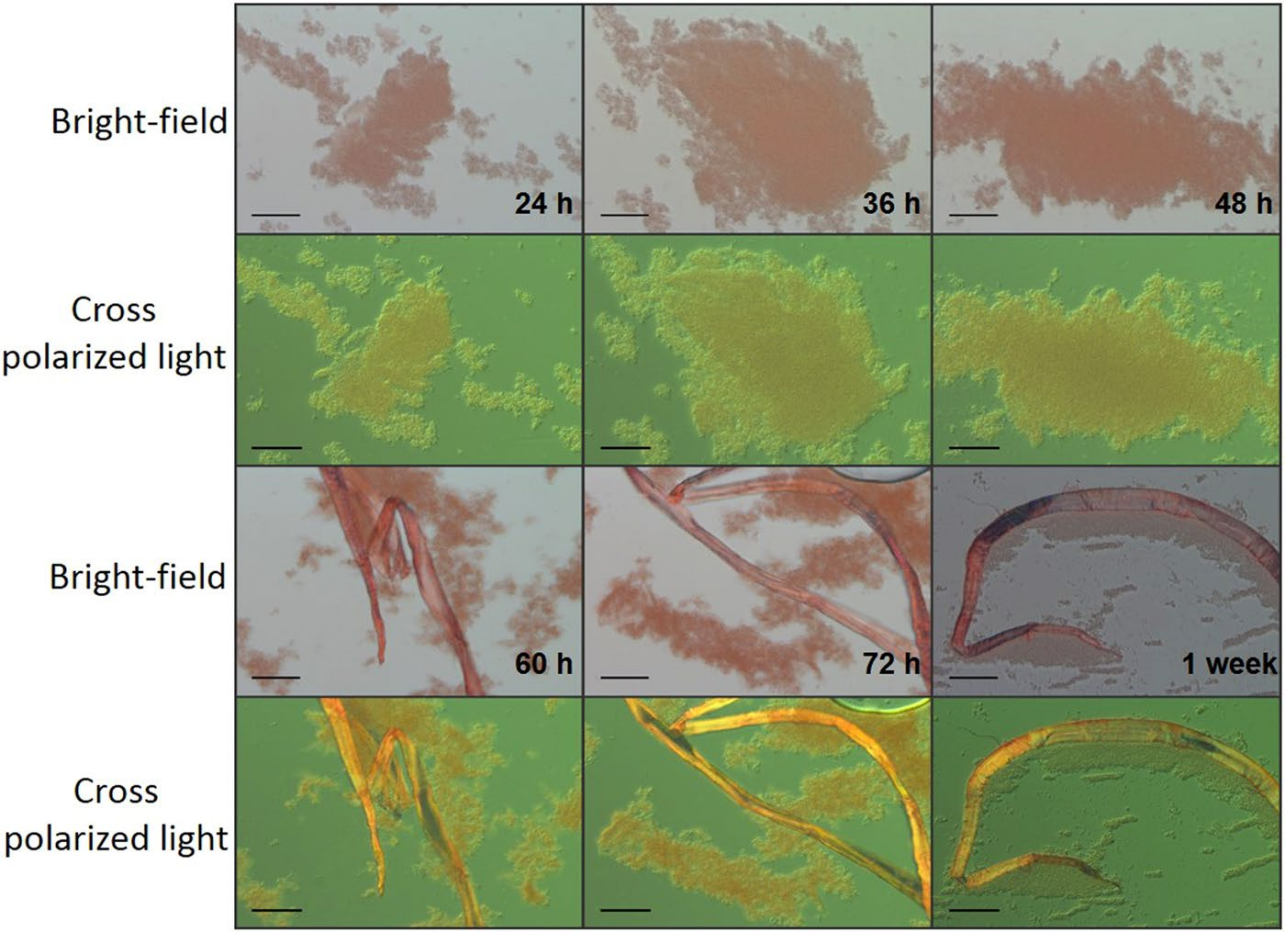
Time course of amyloid formation in *S. mutans* biofilms. Extracellular birefringent rope-like structures were visualized in biofilm material harvested after 60 h of growth as static cultures. Images were captured by bright-field microscopy and under cross-polarizing light filters to visualize Congo Red-induced birefringence at the indicated time points. Scale Bar 50 µm.

P1, WapA, and Smu_63c are constitutively produced by *S. mutans* during both planktonic and biofilm growth. Similar to other bacteria^14,42–44^, amyloid formation by *S. mutans* is biofilm-associated^28^. To assess the time course of amyloid formation within *S. mutans* biofilms, we evaluated static biofilms at 24 h and up to 1 week. CR-induced birefringence was evaluated every 12 h (Fig. 7). Birefringent material was not visible until the biofilms had grown for 60 h.

## Discussion

*S. mutans* is a highly acidogenic organism that accumulates in dental plaque where it clings to tooth surfaces in a tenacious biofilm encased in an ECM composed of glucan and fructan polymers, proteins, and extracellular DNA (eDNA). While glucosyltransferase enzymes and glucan binding proteins are critical for sucrose-dependent bacterial adhesion, cell surface proteins such as P1 and WapA mediate sucrose-independent adhesion to constituents of the salivary pellicle coating tooth surfaces^16,45^. In streptococci and staphylococci, truncation derivatives of several adhesins are now known to polymerize into amyloid fibers potentially facilitated by eDNA^13,17^. The protease resistant amyloid-like material harvested from the ECM of *S. mutans* biofilms exists in an apparent complex with eDNA^8^. The C123 and AgA fragments of P1 and WapA are both capable of amyloid formation^26^. The structure of P1 has been extensively characterized^37,46,47^. The C123 fragment has been shown to interact with cell wall localized-full-length P1^48,49^. Specific amino acid residues involved in that binding have been characterized by NMR^50^. While WapA has not been as widely studied as P1, it also contributes to *S. mutans* cell surface architecture and biofilm formation^45,51^. The N-terminal amyloidogenic AgA fragment is also reactive with type I collagen and fibronectin^52^. The third known amyloid forming protein of *S. mutans*, Smu_63c, is secreted, and a Δ*smu_63c* single mutant demonstrates increased biofilm cell density and genetic competence^26,31^. The functional amyloid Fap of *Pseudomonas aeruginosa* interacts with quorum sensing molecules involved in cell density signaling cascades^19^; however, it is not yet known whether *S. mutans* Smu_63c interacts with competence activating peptides^53^, either in monomeric or amyloid form. Mutants lacking P1, C123, and Smu_63c singly and in combination have been generated, with the triple mutant strain most seriously impaired in biofilm formation^26^. Two of the three amyloidogenic *S. mutans* proteins are linked to the cell surface by sortase. A mutant strain lacking this enzyme does not form amyloid despite producing its substrate proteins in secreted form^28^. Interestingly, three of the eight known amyloidogenic proteins of *Streptomyces coelicolor* are also sortase substrates^54^. A common theme that emerges from these studies is that amyloidogenic bacterial proteins are either surface-associated or secreted, and therefore a part of the extracellular matrix. Given the increasing number of bacterial amyloid forming proteins identified, and the potential factors that may contribute to their ability to polymerize, it is of interest to be able to track amyloid formation specifically within the complex milieu of bacterial biofilms.

When amyloid material produced from each of the three *S. mutans* proteins was visualized by TEM before and after protease digestion, a striking difference in morphology became apparent. Crude amyloid induced by mechanical agitation appeared as mat-like structures, whereas protease digestion of the mats revealed long thin fibers more typical in appearance to that reported for classical disease-related amyloids found in mammals^1,39^. All four amyloidophilic dyes we tested demonstrated increased fluorescence when reacted with amyloid mats or purified amyloid fibers, but not protein monomers. C123, AgA, and Smu_63c amyloid mats and fibers also displayed CR-induced birefringence. Because of the resemblance of the mats to nanocomposites of graphene and amyloid^33^, we considered that they might represent a higher order structure composed of fibers held together by protein monomers and/or prefibrillar protease-sensitive oligomeric intermediates. Mat-like structures were indeed reconstituted when fibers and monomers were incubated without agitation, whereas fibers alone were stable over the 4 week time course of the experiment. Similar to engineered amyloid nanocomposites, the fibers “disappeared” when integrated into the supramolecular structure^33^. The reconstitution of mats in the presence of monomer suggests that the preferred form of *S. mutans* amyloid is as a supramolecular structure, which may exist in equilibrium with monomer and possibly oligomeric intermediates.

Size and shape of amyloid aggregates and kinetics of their formation are affected by physiochemical factors including the environmental surfaces to which they are exposed^55^. Extracellular material present in 5 day old *S. mutans* biofilms was reactive with anti-P1, WapA, and Smu_63c specific antibodies when visualized by immunogold electron microscopy and appeared more mat-like than fibrillar in nature^26^. This suggests that mats represent a naturally occurring amyloid-containing form produced during *S. mutans* biofilm growth. Mat-like amyloid aggregates have also been visualized within biofilm ECM of *Bacillus subtilis* and *Staphylococcus aureus*^17,32^. The observed difference in suspension of amyloid mats compared to purified fibers may confer a biological advantage to an organism such as *S. mutans* that dwells in the aqueous environment of the oral cavity. The encasement of fibrillar amyloid within *S. mutans* ECM was supported by visualization of thin and thick fibers when extracellular biofilm material was separated from whole cells, treated with DNAse, RNAse and proteinase K, and analyzed by TEM^8^.

The ability to form amyloid fibers is not directly related to the primary sequence of any given polypeptide backbone, although prediction programs can suggest possible amyloid forming regions. Nonetheless, amyloid fibers that form from different peptides or proteins share the common feature of high β-sheet content that presents as similar X-ray diffraction patterns of the amyloid aggregates^56^. Analysis of amyloid mats and purified fibers of C123, AgA, and Smu_63c by X-ray fiber diffraction was consistent with a typical stacked cross-β amyloid structure achieved by each protein. Importantly, the observed diffraction patterns of the three different proteins were strikingly similar to one another, as previously reported when amyloids of disparate protein origin have been characterized by this technique^57–60^. The observed patterns also indicate more conventional β-amyloid assemblies for each of the *S. mutans* proteins rather than the unusual cross-α amyloid-like assembly that has been reported for the phenol soluble modulins of *S. aureus*^25^. The diffraction patterns we observed are similar to those reported for synthetic peptides corresponding to the amyloidogenic chaplin proteins of *Streptomyces coelicolor*, as well as to a crude mixture of proteins extracted from the bacterial cell wall of that organism^54^. It is not yet known whether the different chaplin proteins are capable of forming heteroaggregates^54^. Likewise, it is unknown whether *S. mutans* proteins form primarily homo or hetero amyloid aggregates during biofilm growth and this question will be the topic of further study.

While, it has been reported that *S. mutans* amyloid adopts a unique α-sheet structure^61^, this conclusion was based on indirect evidence in which α-sheet peptides comprised of alternating L- and D-amino acids inhibited ThT uptake and fluorescence by *S. mutans* biofilms. Our data caution against using fluorescent dye uptake as a surrogate marker of amyloid fibrilization within biofilms of this organism and demonstrate high background binding of dye directly to the bacterial cells in the absence of amyloid (Figs. 6 and S5). Thus in a cellular context uptake of fluorescent dyes is indicative of *S. mutans* cell number. Because D-amino acids can interfere with bacterial protein synthesis^62^, as well as with growth of early biofilm foci into larger assemblies of cells after initial bacterial attachment^63^, it is critical to substantiate conclusions regarding potential amyloid polymerization mechanisms of *S. mutans* biofilm ECM proteins by biophysical methods other than ThT or CDy11 uptake. CR-induced birefringence appears to be a more specific indicator of amyloid formation in *S. mutans* biofilms. When a time course experiment was performed to identify amyloid in *S. mutans* biofilms, CR-induced birefringent material was not evident until biofilms had been cultured for 60 h. This suggests that amyloid is not present or prevalent early in biofilm development during initial adhesion of the bacterial cells, but rather accumulates within the extracellular matrix as the biofilms age. This may aid in cellular consolidation and accumulation into macrocolonies that appear within mature biofilms^64^.

In addition to amyloid, macromolecules contained within extracellular matrices (ECM) of *Streptococcus mutans* biofilms include extracellular DNA, as well as soluble and insoluble glucan polymers produced in the presence of sucrose by three different glycosyltransferases, GtfB, C, and D (reviewed in Lemos *et al*. 2019)^16^. In *S. mutans*, sucrose-dependent biofilm formation is severely impaired in a triple mutant lacking *gtfBCD*, whereas deletion of genes encoding the sucrose-independent adhesins P1 and WapA has a less obvious impact on sucrose-dependent biofilm formation^26^. In the current study amyloid formation was tracked in biofilms grown without sucrose. However, it was demonstrated previously that amyloid inhibitors impede *S. mutans* biofilm formation whether sucrose is present or not, and that during growth in sucrose mutant strains lacking genes encoding P1 and/or WapA lose sensitivity to biofilm inhibition by small-molecule amyloid inhibitors compared to wild-type cells^26^. This suggests functional cooperation of amyloid forms of P1 and WapA-derived polypepetides with adhesive glucans formed during biofilm development in sucrose. Bacterial amyloids present in biofilm extracellular matrices are reported to confer stability by interacting with both extracellular DNA as well as exopolysaccharides^13^. Taken together, the current results will aid in the design of future studies to investigate amyloid interactions with other ECM components, to evaluate environmental triggers of amyloidogenesis within *S. mutans* biofilms, and to understand potential mechanisms of action of compounds that inhibit both amyloid and biofilm formation in this organism.

## Materials and Methods

### Amyloid induction

Recombinant C123, AgA, and Smu_63c were purified and amyloid induced by mechanical agitation (stirring) as described^26^. Amyloid formation was facilitated in this study by seeding with pre-formed amyloid. Purified proteins were adjusted to 1 mg/mL in 500 µL of 25 mM Tris, 200 mM NaCl, pH 8.0 for C123 and AgA, or 25 mM PO_4_, 300 mM NaCl, pH 4.0 for Smu_63c, and stirred at 4 °C in a 1.5 ml Eppendorf tube using a 10 ×3 mm micro stir bar (Fisher Scientific) for 3–5 days on a Fisher Scientific Isotemp stir plate at 1200 rpm. Next, 3.5 mL of purified recombinant protein (1 mg/mL) was seeded with the 500 µL of preformed amyloid. Samples were stirred as above in 15 mL falcon tubes using octagon Spinbar magnetic stirring bars (25.4 ×8 mm) for 7 days.

### Purification of amyloid fibers

Induced amyloid (1 mg/mL) was treated with proteinase K (PK) at a 10:1 (amyloid:PK) molar ratio. C123 was incubated at 37 °C for 1.5 h, and AgA and Smu_63c were incubated for 3 h. Reactions were stopped by addition of phenylmethylsulfonyl fluoride (PMSF) to 2 mM and incubation for 5 min at room temperature. Samples were ultra-centrifuged at 10 °C for 30 min at 100,000 X g, using a Beckman Optima TLX (TLA-55) rotor. Pellets were resuspended in 1 ml double-distilled water (ddH_2_O), and ultra-centrifugation repeated with resuspension in 1 mL ddH_2_O. Samples were monitored for elimination of residual monomers by SDS-polyacrylamide gel electrophoresis and Western blotting using rabbit polyclonal antisera against AgA or Smu_63c, or the C123 specific monoclonal antibody 6–8C^48^. Residual monomer was eliminated from the C123 sample by additional treatment with 0.1% (v/v) Triton X100 for 15 min at room temperature. To quantify fiber yield, samples were dried overnight under vacuum in tared tubes in a refrigerated vapor trap (Savant RVT 100) followed by weighing with an analytical balance (AL54 Mettler Toledo).

### Transmission electron microscopy

One drop of crude amyloid or purified fiber sample (1 mg/mL in water) was placed under a 100-mesh formvar-carbon coated grid FCF100-Cu-UB (Electron Microscopy Sciences, Hatfield, PA) for five minutes. Grids were transferred to one drop of ddH_2_O for one min and stained by placement over a drop of 2% uranyl acetate for 2 min. Excess uranyl acetate was removed by setting grids in ddH_2_O for two sec. Imaging was done using a Hitachi H7600 transmission electron microscope (Hitachi High Technologies America, Schaumburg, IL). Digital images were acquired using an AMT digital camera. In the case of mat reconstitution, P1-C123 monomer (1 mg/mL) was added to 100 µL of pure fibers (1 mg/mL) to a final concentration of 1% or 10% (w/w) and incubated at 37 °C for 2 or 4 weeks. The negative control consisted of pure fibers (1 mg/mL) without added monomer.

### X-ray fiber diffraction

Induced amyloid and purified fiber samples were prepared in enclosed plastic cuvette chambers as previously reported^23^, with minor modifications. A 5–10 µL drop of each sample was suspended between two 1 mm glass rods dipped in beeswax held in place 1.5 mm apart. The optimal concentrations needed to suspend the drop and allow the protein samples to dry without breaking were determined in pilot experiments. Amyloid mats were suspended at 20 mg/mL, and purified amyloid fibers at 50 mg/mL, in double distilled water. Cuvettes were placed in closed containers with saturated potassium sulfate for purified fibers, or saturated sodium chloride for amyloid mats, to maintain relative humidity at ~97% or ~75%, respectively. Samples were dried for up to two days. Diffraction data were collected at beamline 4-2 of the Stanford Synchrotron Radiation Lightsource (SSRL) at the SLAC National Accelerator Laboratory. The beam was slit collimated to a size of 200 ×200 µm, and data were recorded using a RayonixMX225HE detector with an energy of 11.5 keV placed at a distance of 334.4 mm from the fiber. The program WCEN^65^ was used to analyze the data.

### Fluorescent dye assays

Thioflavin T (ThT) was from Fluka. CDy11, BoDipy-Oligomer (BD-oligo) and MK-H4 were kind gifts from Dr. Young-Tae Chang, Pohang University of Science and Technology, Korea. A 2 mM stock solution of ThT was prepared in TRIS buffer (25 mM TRIS 200 mM NaCl pH 8). Stock solutions of CDy11, BD-oligo, and MK-H4 were 1 mM each in 10% DMSO. For ThT assays, 10 μL of each protein sample (1 mg/mL) were mixed with 70 μL of ddH_2_O and 20 μL of 20 μM of ThT stock solution (final [ThT] = 4 μM). For CDy11, BD-oligo, and MK-H4 assays, 10 μL of each protein sample (1 mg/mL) were mixed with 80 μL of ddH_2_O and 10 μL of 100 μM of dye (final [dye] = 10 μM). Samples were added to 96-well all-black flat-bottom Microfluor plates and incubated in the dark for 15–30 min at room temperature. Fluorescence intensities of dyes were measured using a BioTek Synergy 2 or Synergy H1 spectrophotometer at the following emission and excitation wavelengths: ThT (Ex = 440, Em = 485), CDy11 and BD-Oligo (Ex = 530, Em = 580), and MK-H4 (Ex = 475, Em = 567). All experiments were performed in triplicate with background dye fluorescence subtracted from protein plus dye samples.

### Congo red-induced birefringence

Induced amyloid and purified amyloid fibers of each protein were stained with Congo red (CR) and evaluated for birefringence as described^26,28^. One hundred microliters (1 mg/mL) of each sample were centrifuged at 16,000 x g (Eppendorf centrifuge 5415 R). Pellets were resuspended in 10 μL of 500 µM CR solution in 80% ethanol, and incubated for 30–60 min at room temperature. Visualization was with a Zeiss Scope A1 equipped with a computer-controlled ProgRes C5 Jenoptik inverted camera and crossed polarizing light filters. For static biofilm assays, *S. mutans* strain UA159 was grown overnight in Todd–Hewitt broth with 0.3% yeast extract, diluted 1:100 in biofilm media^66^ containing 0.8% of glucose, and 1 mL aliquots placed in wells of a 24 well plate (24 well Costar Flat bottom with lid). Every 12 hours biofilm material was scraped from the bottom of a well and suspended in the culture supernatant, placed in a 1.5 mL Eppendorf tube, and centrifuged at maximum speed in an Eppendorf centrifuge 5415r. Pellets were stained with CR as above. This experiment was performed twice with triplicate wells analyzed for each condition.

### Microfluidic experiments

With cells growing in microfluidic flow channels, we compared CDy11 uptake by *S. mutans* UA159 wild-type to uptake by a triple mutant (Δ3) lacking P1, WapA, and Smu_63c^26^. Bacteria were grown overnight in brain heart infusion (BHI, BD) at 37 °C in 5% CO_2_. Cells were harvested by centrifugation (1200 X g for 10 min), washed once in BHI, and resuspended in BHI to an OD_600_ of 0.1. In order to de-chain the cells, bacterial suspensions were sonicated for 20 seconds using a Fisher Scientific FB120 sonic dismembrator probe at the 20% amplitude setting, and then loaded into a commercial microfluidic device (IBIDI μ-slide VI), which consists of parallel flow channels that are 400 μm deep and 3.8 mm wide. Fresh BHI was flowed through each channel at 0.06 mL/h for 72 hours and then switched to BHI containing 300 nM CDy11 for 4 hours. Phase contrast and red fluorescence images were collected for each strain using an inverted microscope (Nikon TE2000U) equipped with a Photometrics Prime camera and DsRed filter cube. To evaluate CR-induced birefringence, the wild-type and Δ3 mutant strains were grown overnight in Todd-Hewitt broth (THB), harvested by centrifugation and resuspended to an OD_600_ of 0.1 in THB. Microfluidic channels with low intrinsic birefringence were prepared by washing, drying and autoclaving borosilicate glass capillaries (0.05 mm ×0.05 mm ×50 mm, Wale Apparatus). Capillaries were sealed to clean microbore Tygon tubing with epoxy adhesive, with a Luer-slip connection inserted into the tubing to allow loading of cells into the capillary. Cell cultures were sonicated briefly using a Fisher Scientific FB120 sonic dismembrator probe and then loaded (100 µL of cell culture per channel) into the glass capillaries and allowed to settle for one hour. After that, fresh THB was flowed at 0.06 mLhr^−1^ for 72 hours and switched to THB with 100 μM CR solution^28^ for 3 hours. Capillaries were then mounted onto the stage of the Nikon TE2000U inverted microscope and phase contrast and cross-polarized images in visible light were captured.

## Supplementary information

Supplementary Information.
