# Supplementary material for: Enhanced purification coupled with biophysical analyses shows cross-β structure as a core building block for Streptococcus mutans functional amyloids
Source: Sci Rep. 2020 Mar 20;10:5138. doi: 10.1038/s41598-020-62115-7 (PMC7083922; doi:10.1038/s41598-020-62115-7)
Supplement: Supplementary file 1 — Supplementary Information. [file 41598_2020_62115_MOESM1_ESM.pdf]

Supporting Information

Enhanced purification coupled with biophysical analyses shows cross-β structure as a core building block for *Streptococcus mutans* functional amyloids

Ana L. Barran-Berdon[1], Sebastian Ocampo[1], Momin Haider[3], Joyce Morales-Aparicio[1], Gregory Ottenberg[1], Amy Kendall[2], Elena Yarmola[1], Surabhi Mishra[1], Joanna R. Long[4], Stephen J. Hagen[3], Gerald Stubbs[2], and L. Jeannine Brady[1]*

[1]Department of Oral Biology, University of Florida, Gainesville Florida, USA

[2]Department of Biological Sciences and Center for Structural Biology, Vanderbilt University, Nashville, Tennessee

[3]Department of Physics, University of Florida, Gainesville, Florida, USA

[4]Department of Biochemistry, University of Florida, Gainesville, Florida, USA

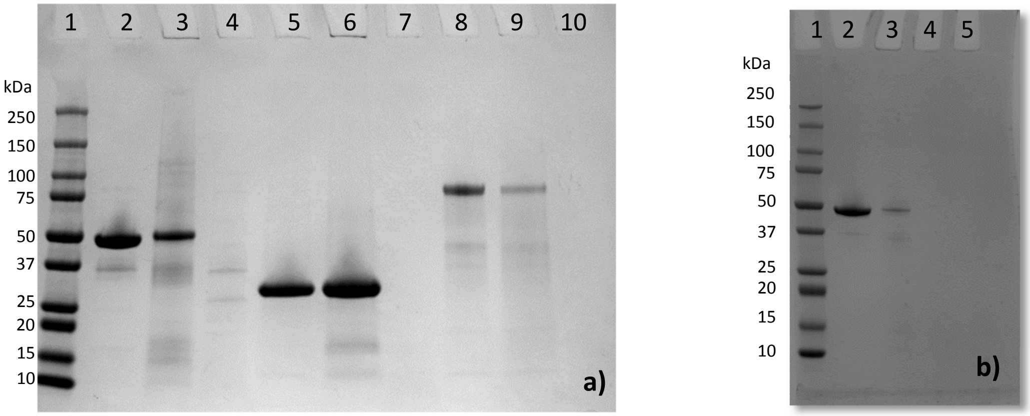

Fig. S1. Elimination of residual protein monomers from induced amyloid material by digestion with proteinase K (PK). a) SDS-polyacrylamide electrophoresis of lane 1, molecular weight standards; lane 2, purified C123 monomer; lane 3, C123 following amyloid induction; lane 4, C123 amyloid after 3 h of PK treatment; lane 5, AgA monomer; lane 6, AgA following amyloid induction; Lane 7, AgA amyloid after 3 h of PK treatment; lane 8, Smu_63c monomer; lane 9, Smu_63c after amyloid induction; lane 10, Smu_63c amyloid after 3 h of PK treatment. b) SDS-PAGE of C123. lane 1, molecular weight standards; lane 2, purified C123 monomer; lane 3, C123 following amyloid induction, Lanes 4 and 5. Supernatant and pellet (purified fibers) fractions following treatment of C123 amyloid material with PK and TritonX100. Approximately 2.5 µg of protein was loaded per well. Images were taken with GeneSys V1.3.7.0 and crop with ImageJ for publication purposes.

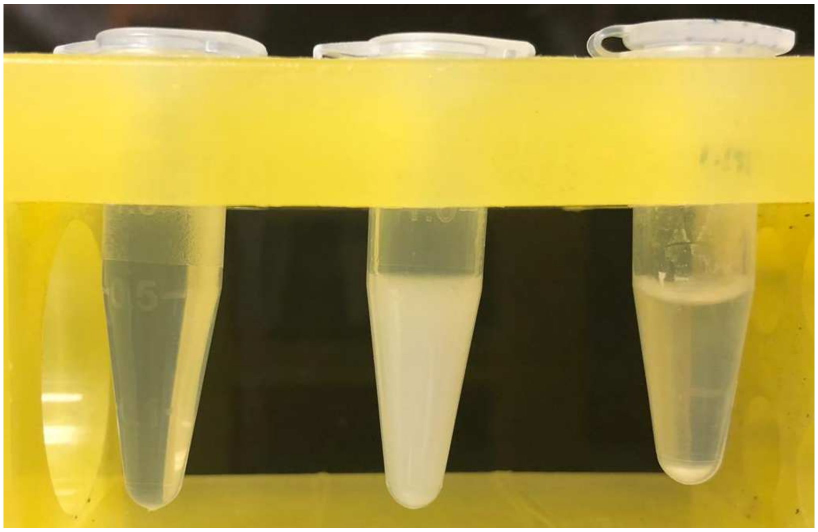

Fig. S2. Left, Solution of purified recombinant P1-C123 monomer; Center, Suspension of P1-C123 after amyloid induction; Right, Pure fibers of P1-C123 following elimination of monomers by digestion with proteinase K and TritonX100.

```
C123            HFHYFKLAVQPQVNKEIRNNNDINIDRTLVAKQSVVKFQL-------KTADLPAGRDETT   53
WapA            ----------------------MKMKRKLLSLVSVL------------------------   14
SMU_63c         ------------------------MSKKFLFNRSVFSNAKGHDVKRKSSKGLVTGIALAG   36
                                    :.:.::   **.

C123            SFVLVDPLPSGYQFNPEATKAASPGFDVTYDNATNTVTFKATAATLATFNADLTKSVATI   113
WapA            -TILLG----AF-WVTK---IVKADQVTNYTNTAS-------------------------   40
SMU_63c         AIVLLG----GS----Q---IASADNVTASENNTTT---SSTAADTDTANSQTVDSTDSD   82
                          :*:.    .     :    .. .  .   * :.

C123            YPTVVGQVLND-------GATYKNNFTLTVNDA---------------------YGIKSN   145
WapA            ------------------------------------------------------------   40
SMU_63c         NSQVTSETVSSKNSTASSEAASESNEAETNNDATASESADQSDDELSDETTSNEAQVKSQ   142

C123            VVRVTTPGK---PND-PDNPNNN------------YIKPTKVNKNENGVVIDGKTVLAGS   189
WapA            ------------------------------------------ITKSDGTALSND------   52
SMU_63c         SVNALESAKYDKDDDEEEEEVNEYKEDDKSEKADIKFDNTGVKTTSSGVNIDGKNITITS   202
                                     ....*. :...

C123            TNYYELTWDLDQYKNDRSS---------------------------------------A   209
WapA            ---------------------------PSKAVNYWEPLSFS-----------NSITFPDE   74
SMU_63c         AGTYTITGSASGYSISVADKVTDTVKLKLDAVNLTDSTLYSSRDLDIKVLSDSSISSSLK   262

C123            DTIQK-GFYYVDDYPEEALELRQDLVKITDANGNEVTGVSVDN-YTNLEAA---------   258
WapA            VSIKAGDTLTIKLPEQ--L-------QFTT--------------ALTFD-----------   100
SMU_63c         NTIETGGALYISSKKKSGL-------KVTSTAGHAIKANSLEADKVTLELSSTAKDGINA   315
                   :*:  .   :.   :  *       :.*                 .::

C123            -------PQEIRDVLSKAGIRPKGAFQIFRADNPREFYDTYVKTG-----IDL-------   299
WapA            ------------VMHTNGQLAGKATTDPNTGEVTVTFTDIFEKLP-NDKAMTLNFNAQLN   147
SMU_63c         TSNVSIKKSNVTISAEDDGIQAEDNTDVNSGDIQIK--DSIVKITSTSKGITA-------   366
                         .  :  :   :   .:      *   *       :

C123            ------KIVSPMVVKKQMGQTGGSYENQ-AYQIDFGNG--------YA----SNIVINNV   340
WapA            HNNISIPGVVNFNYNN---VAYSSYVK--DKDI--TPISPDVNKVGYQDKSNPGLIHWKV   200
SMU_63c         NDEITVKGSTFITITSGSEGIEGRYVNLKKGQITINAGDDAINATEWTTKDDADLSHLKN   426
                   :  ..      . * :    :*             :      .:   :

C123            PKINPKKD----VTLTLDPADTNNVDGQTIPLNTVFNYRLIGGII----PANHSEELFEY   392
WapA            LINNKQGAIDNLTLTDVVGEDQ-EIV-KDS-LVAARLQYIAGDDVDSLDEAASRPYAEDF   257
SMU_63c         SK----KDIENEVAIVISGANISGIG-KDDGVDSNGNLYITDGSL-------KIQSITDY   474
                   .   :   :   :  :   : :     : .. :             ::

C123            NFYDDYDQTGDHYTGQYKVFAKVDITLKNGVIIKSGTELTQYTTA----------EVDTT   442
WapA            SKN--------------VTYQTNDLGLTTGFTYT--------------------------   277
SMU_63c         SSAIDYDGTGFASGG--TTWAIGHMGFAQGFSKGTK---QAYIAAIVSGLAGDTITITDS   529
                           .                 .:   .: :  *.

C123            KGAITIKFKEAFLRSVSIDSAFQAESYIQMKRIAVGT-F-------ENTYINTV------   488
WapA            ----------------------------------------IPGSSNNAIFISYTTRLTSS   297
SMU_63c         KGHIVAKTK--------ADVDFDHVVFS-NKTIKAGKTYTVTTSDGHKAVIKATKDT-TT   579
                                       .   *. .

C123            --NG----------------VTYSS--------- 495
WapA            QSAGKDVSNT-IAI--SGNNINYSNQTGY----- 323
SMU_63c         HPSGRHVSKDTVPLLPNGHHPAFPGNGTPPNDKN 613
                 *                  : .
```

Fig S3. Clustal Omega alignment of C123, WapA, and Smu_63c amino acid sequences. Identical residues are marked by an asterisk and homologous residues are marked by a dot.

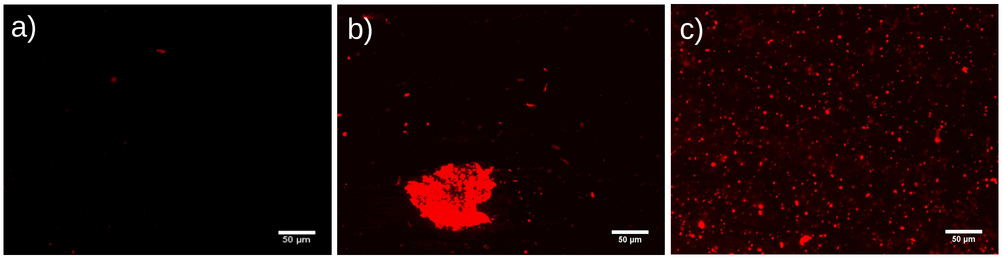

Fig S4. Fluorescence microscopy following CDy11 staining of P1-C123 monomer (a), after amyloid induction (b), and after fiber purification (c). Scale bar 50 µm. Fifty microliters of P1-C123 monomer, amyloid or fibers (1 $mgmL^{-1}$) were treated with 0.5 µL of 100 µM of CDy11 and incubated for 30 min at room temperature. Images were captured with a Zeiss Axiovert 200M microscope equipped with a Zeiss AxioCam MRm camera using 40x magnification.

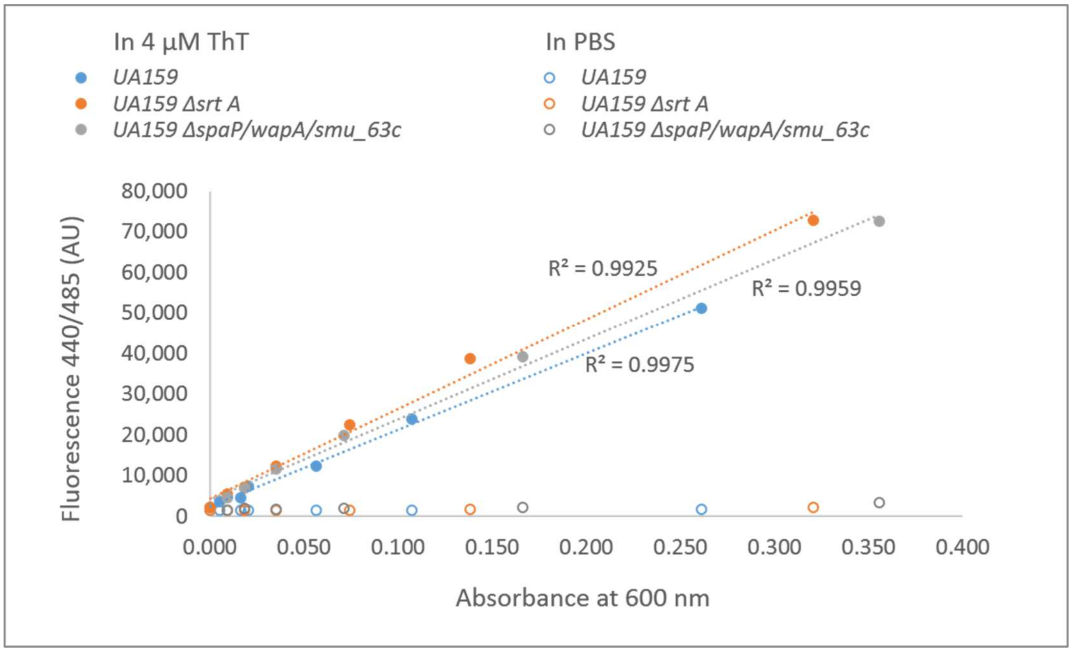

Fig. S5. ThT fluorescence intensity correlates with the cell mass for the wild type and amyloid-deficient strains. Overnight cultures of *UA159*, *UA159Δsrt A,* and *UA159* lacking P1 (encoded by *spaP*), WapA, and Smu_63c grown in THYE media (Todd–Hewitt broth with yeast extract) were centrifuged for 10 min at 16,100 g (Eppendorf mini-centrifuge 5415R), and re-suspended in the same volume of PBS. Then, serial 2-times dilutions in PBS were made out of each culture (last sample in each dilution series was pure PBS). Ninety microliters of each cell dilution was mixed with ether 10 µl of PBS, or with 10 µl of 40 µM ThT (diluted in PBS, final ThT concentration 4 µM) in the wells of a Corning 96 well plate (Product # 3595). Then absorbance at 600 nm and fluorescence (excitation at 440 nM and emission at 485 nm) were read using a Biotek Synergy plate reader.
